# Photocatalytic activity and mechanism of YMnO_3_/NiO photocatalyst for the degradation of oil and gas field wastewater

**DOI:** 10.3389/fchem.2024.1408961

**Published:** 2024-05-01

**Authors:** Jiang Zhu, Xiaoyi Cheng, Yajing Cui, Feng Chen

**Affiliations:** ^1^ Yangzhou Inspection and Testing Center, Yangzhou, China; ^2^ School of Materials Science and Engineering, Suzhou University of Science and Technology, Suzhou, China; ^3^ Key Laboratory of Environmental Functional Materials in Jiangsu Province Universities, Suzhou University of Science and Technology, Suzhou, China

**Keywords:** hydrothermal method, YMnO3, NiO, oil and gas field wastewater, photocatalytic activity

## Abstract

One-step hydrothermal method has been used to synthesize YMnO_3_@NiO (YMO@NO) photocatalysts with high photocatalytic activity for the degradation of oil and gas field wastewater under simulated solar irradiation. Through various characterization methods, it has been confirmed that the YMO@NO photocatalyst comprises only YMO and NO, without any other impurities. The microstructure characterization confirmed that the YMO@NO photocatalyst was composed of large squares and fine particles, and heterojunction was formed at the interface of YMO and NO. The optical properties confirm that the YMO@NO photocatalyst has high UV-vis optical absorption coefficient, suggesting that it has high UV-vis photocatalytic activity. Taking oil and gas field wastewater as degradation object, YMO@NO photocatalyst showed the highest photocatalytic activity (98%) when the catalyst content was 1.5 g/L, the mass percentage of NO was 3%, and the irradiation time was 60 min. Capture and stability experiments confirm that the YMO@NO photocatalyst is recyclable and electrons, holes, hydroxyl radicals and superoxide radicals play major roles in the photocatalysis process. Based on experiments and theoretical calculations, a reasonable photocatalytic mechanism of the YMO@NO photocatalyst is proposed.

## 1 Introduction

The development and utilization of fuel oil is essential, regardless of the current economic development or the outbreak of war. In the process of fuel oil development and utilization, there will be a large number of oil and gas field wastewater, which will greatly pollute the natural environment if not effectively treated (Ahmadun et al., 2009). The main component of oil and gas field wastewater is petroleum hydrocarbon, so degrading petroleum hydrocarbon in oil and gas field wastewater by special means becomes the key to reduce environmental pollution (Rabahi et al., 2019). At present, the degradation of oil and gas field wastewater has gradually become the focus of environmental pollution, which makes researchers focus on pioneering research. Photocatalysis technology has become the key technology to solve the pollution of oil and gas field wastewater. It has been widely favored by researchers to make full use of the clean energy of sunlight to degrade the oil and gas field wastewater.

It is well known that photocatalytic technology is particularly important to select the right type of photocatalyst when degrading oil and gas field wastewater. Yttrium manganate (YMnO_3_, YMO) is a common semiconductor photocatalyst, which has a wide range of applications in dyes, antibiotics, carbon dioxide reduction and other fields due to its high thermal and chemical stability, high charge transfer and separation efficiency (Wang et al., 2011; Peng et al., 2017; Kumar et al., 2019; Munisha et al., 2023a; López-Alvarez et al., 2023). In spite of this, researchers are still taking active and effective measures to improve the photocatalytic activity of YMO. 1) Activation ions were introduced into YMO to improve its photocatalytic activity (Zhang et al., 2011; Munisha et al., 2023b). 2) The modification of metal particles on the surface of YMO enhances its active site, thus enhancing the catalytic activity of YMO (González-Castaño et al., 2021). 3) The photocatalytic activity of the system was enhanced by introducing another semiconductor material to form the YMO-base heterojunction (Cao et al., 2018; Wu et al., 2019; Wang and Song, 2020; Wang and Tian, 2020; You et al., 2020; Wang et al., 2022; Zhang et al., 2022; Li et al., 2023; Wang et al., 2023; Yulizar et al., 2023; Dang et al., 2024). The construction of heterojunction is an effective method to improve the photocatalytic activity of YMO. After coupling NiO (NO) with YMO to form YMO@NO photocatalyst by the polyacrylamide gel method, the photocatalytic activity of YMO can be effectively enhanced to degrade methyl orange dye (Wang and Song, 2020).

It is worth noting that the target products synthesized by different preparation methods will have different surface morphologies, which will help to obtain semiconductor materials with better performance (Wang et al., 2011; Wu et al., 2019; You et al., 2020; Dang et al., 2024). Hydrothermal method is an effective method to control the surface morphology of semiconductor photocatalysts by using surfactants. Simultaneously, in the field of photocatalysis, there is too much work focused on photocatalyst degradation of dye wastewater, refractory pollutant and pharmaceutical wastewater. However, no researchers have used one step hydrothermal method to synthesize YMO@NO photocatalyst, which will make the synthesis of YMO@NO photocatalyst in one step hydrothermal method and its application in the degradation of oil and gas field wastewater significant.

In this paper, one-step hydrothermal method was proposed to synthesize the YMO@NO photocatalyst, and its photocatalytic activity for the degradation of oil and gas field wastewater was studied. The effects of catalyst content, irradiation time and mass percentage of NO on the photocatalytic activity of YMO@NO photocatalyst have been deeply explored. Based on stability experiment, trapping experiment and theoretical calculation, the photocatalytic mechanism of YMO@NO photocatalyst is proposed.

## 2 Experimental detail

### 2.1 Synthesis of YMO@NO photocatalysts

YMO@NO photocatalysts were synthesized according to the mass ratios of YMO to NO of 99 : 1, 97 : 3 and 95 : 5. The photocatalysts with the above mass percentage were named YMO@NO 1%, YMO@NO 3% and YMO@NO 5%, respectively. According to the molar ratio of Y: Mn is 1 : 1, appropriate amount of yttrium nitrate and manganese acetate are weighed and added to 50 mL of deionized water. Subsequently, 10 g of urea was weighed and added to the precursor solution, accompanied by magnetic agitation until all reagents were completely dissolved. Nickel nitrate is weighed according to different mass ratios of YMO: NO and added to the above solution. After the nickel nitrate is completely dissolved, the reaction solution is transferred to a reactor lined with Teflon. Tighten the reactor and place it in a drying oven at 220°C for 48 h. After the reactor is cooled, the supernatant is poured away and cleaned twice with hydrochloric acid. Subsequently, it was repeatedly cleaned with deionized water 5 times and dried in a drying oven at 120°C for 24 h to obtain the target product.

### 2.2 Characterization of YMO@NO photocatalysts

The phase structure of the YMO@NO 1%, YMO@NO 3% and YMO@NO 5% was characterized using a Philips diffractometer model PW1800 X-ray powder diffraction (XRD) with the X-ray source of Cukα with 1.541 nm wavelength. The vibrational modes of the YMO@NO 1%, YMO@NO 3% and YMO@NO 5% were measured by a JASCO 640 plus infrared spectrometer with the measured wavenumber of 400–4,000 cm^-1^. Chemical binding energy of the YMO@NO 1%, YMO@NO 3% and YMO@NO 5% were obtained by a Multilab 2000 type X-ray photoelectron spectrometer (XPS) with the operating at 10 kV and 10 mA. The microstructural of the YMO@NO 1%, YMO@NO 3% and YMO@NO 5% were observed by an FEI Inspect S50 scanning electron microscopy (FE-SEM) and Stereo-scan LEO 440 transmission electron microscope (TEM). The ultraviolet-visible absorption spectra of the YMO@NO 1%, YMO@NO 3% and YMO@NO 5% were obtained by PG (UK) instrument T80 UV-visible spectrophotometer. The photoluminescence spectra of the YMO, NO, and YMO@NO 3% photocatalysts were measured by a fluorescence spectrophotometer. Mott- Schottky (M-S) curves of YMO and NO were obtained by an electrochemistry workstation with three electrodes.

### 2.3 Photocatalytic experiments of YMO@NO photocatalysts

There are a lot of pollutants in the wastewater of oil and gas field. The important parameter to judge the pollutants is the chemical oxygen demand (COD). The degradation behavior of oil and gas field wastewater by photocatalyst can be judged by measuring its COD content. Before the experiment, prepare 100 mL of oil and gas field wastewater and 0.05–0.25 g photocatalyst, and put the photocatalyst into the oil and gas field wastewater. In the lighting process, a 500 W xenon lamp is used as a light source to emit simulated sunlight. The initial COD value (COD_0_) of the oil and gas field wastewater is 531 mg/L. The standard iodine titration method is used to test COD. The excess iodine solution is used to react with petroleum hydrocarbon in oil and gas field wastewater under acidic condition. The residual amount of iodine is determined by titrating the excess iodine solution, and the COD value is calculated. When lighting is performed, the reaction solution is taken every 10 min. The COD value in the reaction solution measured after each sample is recorded as COD_t_. The percentage of degradation is defined as ((COD_t_-COD_0_)/COD_0_)×100%. To perform capture experiments, the potassium sulfate (K_2_SO_4_), disodium ethylene diamine tetraacetic acid (EDTA-2Na), isopropyl alcohol (IPA), and benzoquinone (BQ) are used as trapping agent to capture electrons, holes, hydroxyl radicals, and superoxide radicals, respectively. Before the capture experiment, 1 mmol/L of the capture agent was added to the reaction solution, and the remaining experiments were consistent with the photocatalytic experiment.

## 3 Results and discussions

### 3.1 XRD analysis

**FIGURE 1:**
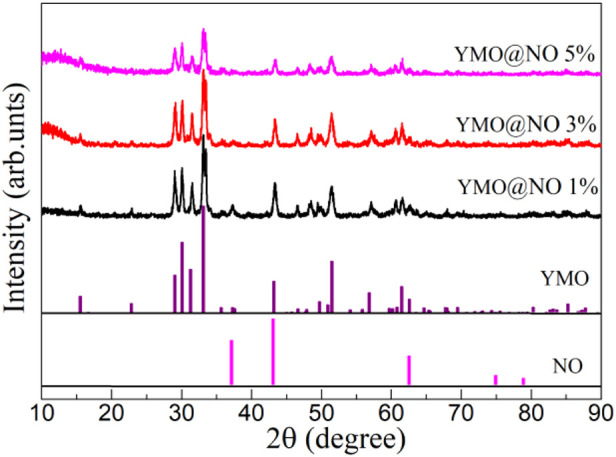
XRD patterns of YMO@NO 1%, YMO@NO 3% and YMO@NO 5%.

The fabricated YMO@NO 1%, YMO@NO, 3%, and YMO@NO 5% photocatalysts have been studied using the X-ray diffraction technique. Figure 1 depicts the XRD patterns of YMO@NO 1%, YMO@NO 3% and YMO@NO 5%. It can be seen from all XRD diffraction patterns that the YMO@NO samples all contain the diffraction peaks of YMO with the standard PDF#70–4,962 and NO with the standard PDF#89–7130. The diffraction peak of NO overlaps with that of YMO, and the intensity of YMO@NO decreases with the increasing of NO content. The evidence that NO coexists with YMO@NO photocatalyst requires further characterization by FTIR, XPS and HRTEM in detail.

### 3.2 FTIR analysis

**FIGURE 2:**
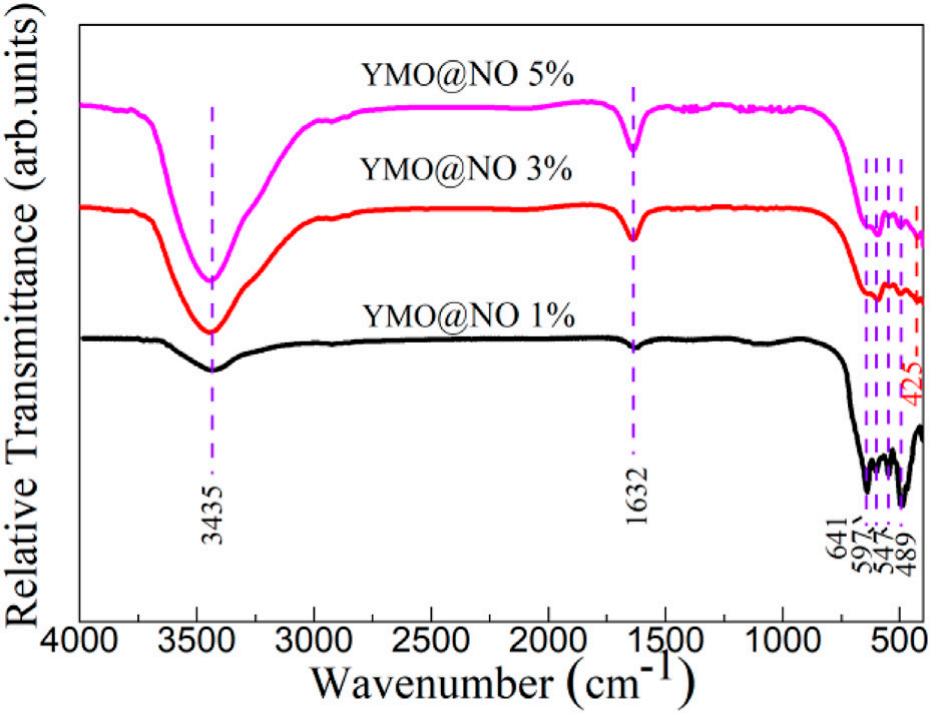
FTIR spectra of YMO@NO 1%, YMO@NO 3% and YMO@NO 5%.

The vibration modes present in YMO@NO 1%, YMO@NO 3% and YMO@NO 5% photocatalysts were investigated through FTIR technique in the frequency range of 400–4,000 cm^-1^. Figure 2 depicts the FTIR spectra of YMO@NO 1%, YMO@NO 3% and YMO@NO 5%. The H-O stretching vibration and H-O-H bending vibration are responsible for the characteristic peaks at 3435 and 1632 cm^-1^ for all samples, respectively (Kumar et al., 2019; Dang et al., 2024). The presence of the above two characteristic peaks in YMO@NO samples confirms that the samples adsorbed small amounts of water molecules during storage. In the low wave number segment, the characteristic peaks of 641, 597, 547 and 489 cm^-1^ appear in YMO@NO 1% photocatalyst. The peaks at 597, 547 and 489 cm^-1^ can be ascribed to the Mn-O stretching vibration, Y-O stretching vibration and Mn-O-Mn bending vibration, respectively (Kumar et al., 2019; Dang et al., 2024). As Iliev and colleagues’ (Iliev et al., 1997) calculation, there is no mode that the peak at 641 cm^-1^ can be assigned to in this study due to the YMO in the YMO@NO 1% photocatalyst. With the increase of NO content, a new characteristic peak at 425 cm^-1^ could be observed for the YMO@NO 3% and YMO@NO 5% photocatalysts. This new characteristic peak can be attributed to the Ni-O bond in NO (Wei et al., 2009; Rahdar et al., 2015). This result confirms that NO exists in YMO@NO photocatalyst.

### 3.3 XPS analysis

**FIGURE 3:**
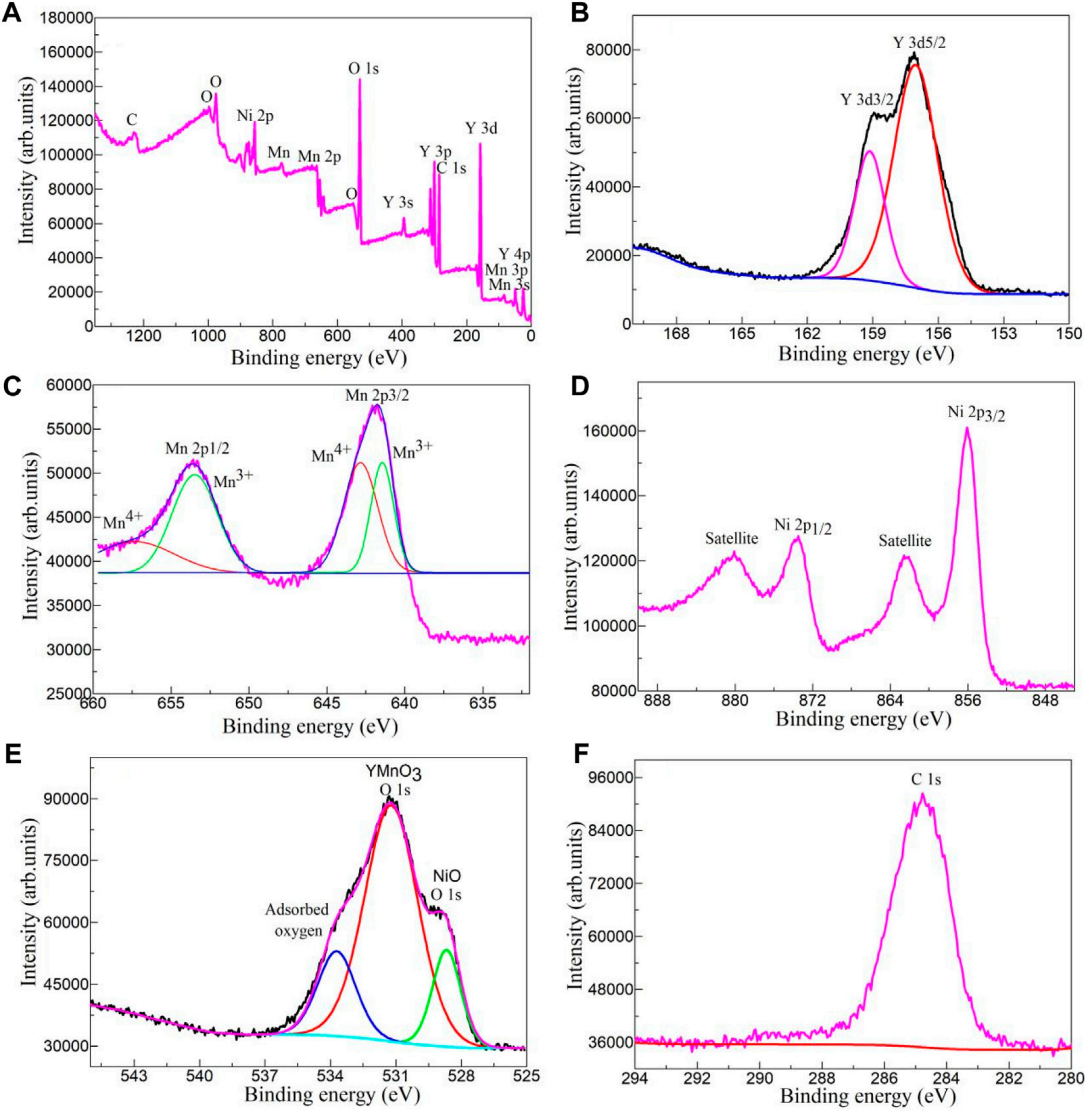
**(A)** XPS survey spectrum, high resolution **(B)** Y 3d, **(C)** Mn 2p, **(D)** Ni 2P, **(E)** O1s and **(F)** C1s spectra of YMO@NO 3%.

X-ray photoelectron spectroscopy (XPS) has been used to study the chemical composition and chemical state of YMO@NO 3% photocatalyst. Figure 3A depicts the XPS survey spectrum YMO@NO 3% photocatalyst. The total survey spectrum in Figure 3A exhibits the signal of main elements including C, O, Ni, Mn, and Y. The results show that the YMO@NO 3% photocatalyst has no other impurity elements because of the C element is the calibration peak of the XPS instrument. The high resolution Y 3d spectrum of YMO@NO 3% photocatalyst as depicted in Figure 3B. Double peaks of Y 3d5/2 and Y 3d3/2 are present in the spectrum at 157.10 and 159.13 eV, respectively. The high resolution Mn 2p spectrum of the YMO@NO 3% photocatalyst is depicted in Figure 3C, in which the Mn 2p3/2 and Mn 2p1/2 spin-orbit doublet peaks are observed at 641.46 and 653.48 eV of Mn^3+^, 642.81 and 657.37 eV of Mn^4+^, respectively. Mn^3+^ ions in YMO can promote its trapping of holes to form Mn^4+^ ions, which is consistent with the conclusion in the literature (Chen et al., 2020; Mukesh et al., 2020; Wan et al., 2023). Four characteristic peaks can be identified as NO as depicted in Figure 3D due to the high resolution spectrum of Ni 2p in YMO@NO 3% photocatalyst. The high resolution O1s spectrum of YMO@NO 3% as depicted in Figure 3E. The high resolution O 1s spectrum of YMO@NO 3% photocatalyst was divided into three obvious characteristic peaks at 533.72, 531.23, and 528.67 eV, which were mainly composed of the adsorbent oxygen, the lattice oxygen of YMO and the lattice oxygen of NO, respectively. [Paul et al., 2020 (Zhang and Wang, 2017),] Figure 3F depicts the high resolution C1s spectra of YMO@NO 3%. In Figure 3F, an characteristic peak at 284.81 eV can be observed due to the self-calibration of instrument. This result further confirms that the YMO@NO 3% photocatalysts contain NO.

### 3.4 Microstructural analysis

**FIGURE 4:**
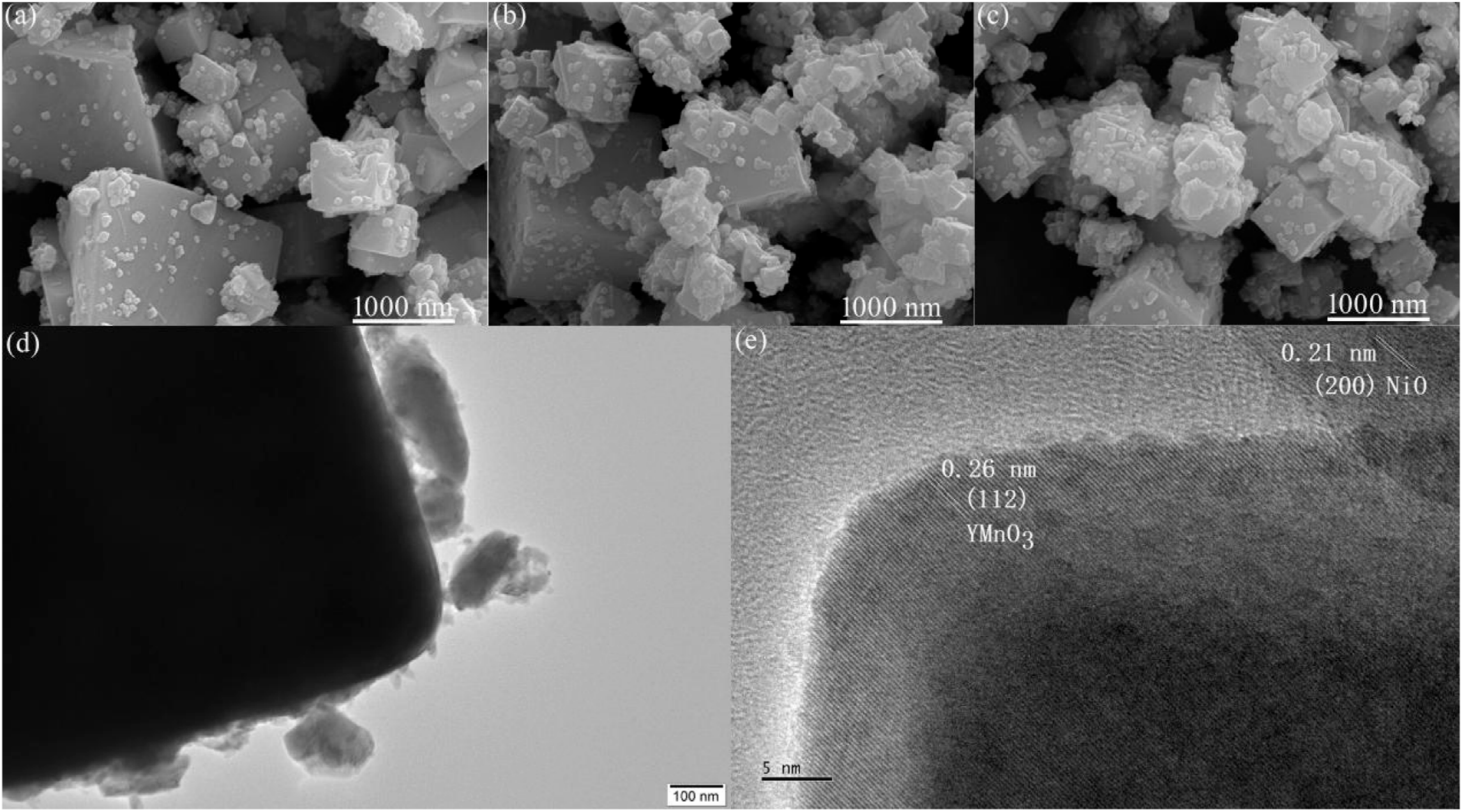
SEM images of **(A)** YMO@NO 1%, **(B)** YMO@NO 3% and **(C)** YMO@NO 5%. **(D)** TEM and **(E)** HRTEM images of YMO@NO 3%.

The surface morphology of the YMO@NO 1%, YMO@NO 3% and YMO@NO 5% photocatalysts was observed via SEM observation. Figures 4A–C depict the SEM images of the YMO@NO 1%, YMO@NO 3% and YMO@NO 5% photocatalysts. All samples are composed of large irregular cuboids and fine particles. With the increase of NO content, the number of fine particles gradually increased. To further explore the details of the surface topography of the YMO@NO 3% photocatalyst, TEM image of the YMO@NO 3% photocatalyst as depicted in Figure 4D. TEM images show large chunks about 2 μm in diameter, while fine particles are less than 100 nm. This result is consistent with that observed by SEM. Figure 4E depicts the HRTEM images of YMO@NO 3% photocatalyst. The HRTEM image of YMO@NO 3% photocatalyst in Figure 4E exhibited the lattice fringes of 0.26 and 0.21 nm, matching well with the (112) of YMO and (200) of NO, respectively. This result once again confirmed the existence of NO in YMO@NO photocatalyst.

### 3.5 Optical properties

**FIGURE 5:**
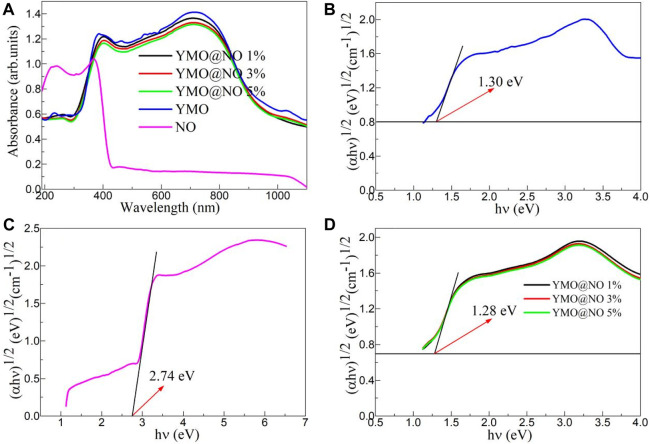
**(A)** Ultraviolet-visible absorption spectra of the YMO, NO, YMO@NO 1%, YMO@NO 3% and YMO@NO 5%. The E.g., values of **(B)** YMO, **(C)** NO and **(D)** YMO@NO 1%, YMO@NO 3% and YMO@NO 5%.

The ultraviolet-visible absorption spectra of the YMO, NO, YMO@NO 1%, YMO@NO 3% and YMO@NO 5% photocatalysts were studied by an ultraviolet-visible spectrophotometer. Figure 5A depicts the ultraviolet-visible absorption spectra of the YMO, NO, YMO@NO 1%, YMO@NO 3% and YMO@NO 5% photocatalysts. For YMO, YMO@NO 1%, YMO@NO 3% and YMO@NO 5% photocatalysts, the absorption coefficients showed three significant increases with the increasing wavelength, mainly in the ranges of 190–270, 300–380, and 450–700 nm. YMO demonstrated the highest optical absorption coefficient. However, NO exhibits high UV optical absorption coefficients at 190–400 nm, and optical absorption coefficients remain essentially constant at 400–1100 nm. When NO was introduced into YMO, the optical absorption coefficient of YMO only decreased slightly. The high optical absorption coefficient in the range of 300–900 nm implies that the YMO@NO photocatalyst has a high visible light response capacity.

The Tauc relationship between (αhν)^1/2^ and hν for the YMO, NO, YMO@NO 1%, YMO@NO 3% and YMO@NO 5% photocatalysts can be described by Eq. 1.

$${\left(\alpha h\nu \right)}^{n}=A\left(h\nu -E_{g}\right)$$ (1)

Where, h is the Planck constant, ν is the frequency, A is a constant and E.g., is the optical band gap (E.g.,) values of the YMO, NO, YMO@NO 1%, YMO@NO 3% and YMO@NO 5% photocatalysts. The (αhυ)^1/2^ *versus* hυ extrapolated to α = 0 gives the E.g., values of the YMO, NO, YMO@NO 1%, YMO@NO 3% and YMO@NO 5% photocatalysts as depicted in Figures 5B–D. The E.g., values of YMO and NO are 1.30 and 2.74 eV, respectively. Generally, two different types of semiconductors coupled together to form a heterojunction do not change the E.g., value of the main lattice phase. In this experiment, a similar phenomenon was observed, with E.g., values of 1.28 eV for all photocatalysts.

### 3.6 Photocatalytic activity

**FIGURE 6:**
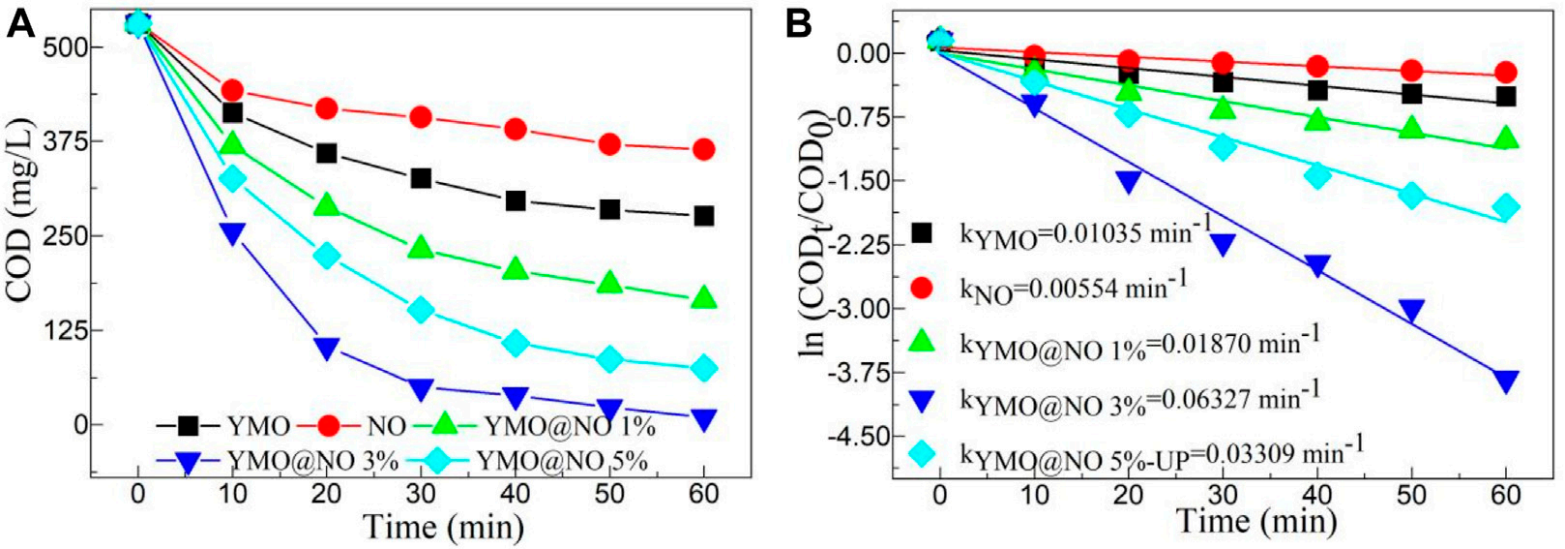
**(A)** COD value decline with time and **(B)** first order kinetic model of the YMO, NO, YMO@NO 1%, YMO@NO 3% and YMO@NO 5%.

According to reports, many different types of petroleum hydrocarbons are present in the wastewater from oil and gas fields. After the degradation of petroleum hydrocarbons, it is necessary to measure the COD value in the reaction solution to understand whether the photocatalyst plays an important role. Figure 6A depicts the COD value decline with time of the YMO, NO, YMO@NO 1%, YMO@NO 3% and YMO@NO 5% photocatalysts. To perform comparative experiments, pure YMO and NO were also selected as photocatalysts to perform degradation experiments. With the increase of irradiation time, the COD value of all samples decreased, indicating that the photocatalyst played an important role in the photocatalytic degradation of oil and gas field wastewater. YMO@NO 3% photocatalyst showed better degradation ability than other photocatalysts.

The first-order kinetic model can effectively reflect the degradation rate of photocatalyst and is often used to describe the degradation behavior of photocatalyst. Figure 6B depicts the first order kinetic model of the YMO, NO, YMO@NO 1%, YMO@NO 3% and YMO@NO 5%. As can be seen from the figure, ln (COD_t_/COD_0_) shows a high linear dependence on time t. The slope is a first-order kinetic constant k). The k values of the YMO, NO, YMO@NO 1%, YMO@NO 3% and YMO@NO 5% photocatalysts are 0.01035, 0.00554, 0.01870, 0.06327, and 0.03309 min^-1^, respectively. There are 6.11 times the degradation rate of the YMO@NO 3% photocatalyst compared to that of YMO and 11.42 times the rate of NO. The results show that the YMO@NO 3% photocatalyst has a high degradation rate in the degradation of oil and gas field wastewater.

### 3.7 Effect of catalyst content on photocatalytic activity

**FIGURE 7:**
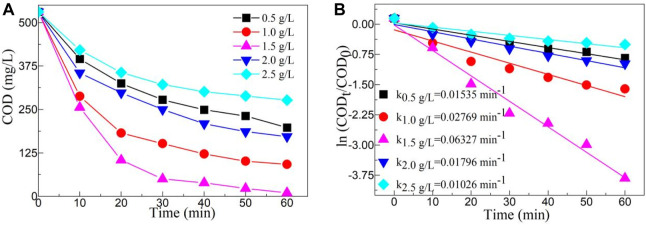
**(A)** Effect of catalyst content on COD value of YMO@NO 3%. **(B)** First-order kinetic models with different contents of YMO@NO 3%.

The photocatalytic activity of a catalyst is greatly influenced by its content. Figure 7A depicts the effect of catalyst content on COD value of YMO@NO 3% photocatalyst. Similarly, regardless of catalyst content, COD value decreases with the increasing irradiation time. The degradation rate of YMO@NO 3% photocatalyst increased with the increasing of catalyst content when the catalyst content increased from 0.5 g/L to 1.5 g/L. With the increase of catalyst content, the surface active site of catalyst was effectively utilized. The catalyst content increase resulted in a further decrease in the degradation rate of the YMO@NO 3% photocatalyst. When the catalyst content is too high, the path length of light entering the oil and gas field wastewater is long, and the active species in the reaction solution decreases rapidly, thus reducing the photocatalytic activity of the YMO@NO 3% photocatalyst (Liu X. et al., 2021; Durán et al., 2022). Combined with the experimental results, the optimum content of YMO@NO 3% photocatalyst for the degradation of oil and gas field wastewater is 1.5 g/L.

Figure 7B depicts the first-order kinetic models with different contents of YMO@NO 3% photocatalyst. Similarly, ln (COD_t_/COD_0_) also has a strong linear relationship with time t. The k values of different contents of YMO@NO 3% photocatalyst including 0.5, 1.0, 1.5, 2.0 and 2.5 g/L are 0.01535, 0.02769, 0.06327, .01796 and 0.01026 min^-1^, respectively. The results further confirmed that the optimum catalyst content of YMO@NO 3% photocatalyst for the degradation of oil and gas field wastewater is 1.5 g/L.

### 3.8 Cyclic stability and capture experiments

**FIGURE 8:**
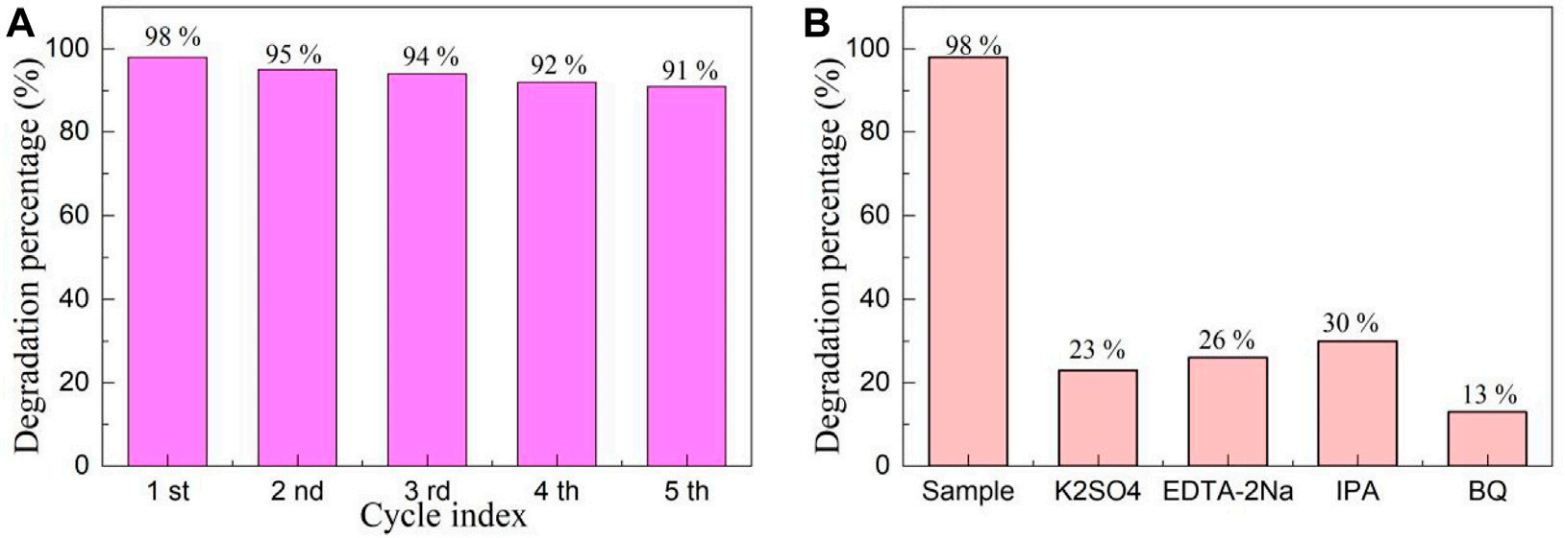
**(A)** Cyclic stability and **(B)** capture experiments of YMO@NO 3%.

The recycling of photocatalyst is crucial for evaluating its industrial application. Figure 8A depicts the cyclic stability experiments of YMO@NO 3% photocatalyst. After the first photocatalytic experiment is performed, the extracted reaction solution and the remaining reaction solution should be mixed together for centrifugation, filtration, drying and sintering to remove contaminants to obtain the photocatalyst required for the cyclic experiment. After 5 cycles of the experiment, it was found that the degradation percentage of YMO@NO 3% photocatalyst only decreased from 98% to 91%. The main reason for the slight decrease in degradation percentage is the loss of photocatalyst in the process of recovery.

Figure 8B depicts the capture experiments of YMO@NO 3% photocatalyst. Electrons, holes, hydroxyl radicals, and superoxide radicals were trapped by using K_2_SO_4_, EDTA-2Na, IPA, and BQ, respectively. The only difference between the capture experiment and the photocatalytic experiment is that the corresponding trapping agent needs to be added during the execution of the capture experiment. When K_2_SO_4_, EDTA-2Na, IPA and BQ were added, the degradation percentage of YMO@NO 3% photocatalyst decreased significantly. However, after adding IPA, the degradation percentage of YMO@NO 3% photocatalyst reached 30%, indicating that hydroxyl radical played a weaker role than electron, hole and superoxide radical in the whole photocatalytic process. This conclusion is helpful to explore the photocatalytic mechanism of YMO@NO 3% photocatalyst.

### 3.9 Photochemical evaluation

**FIGURE 9:**
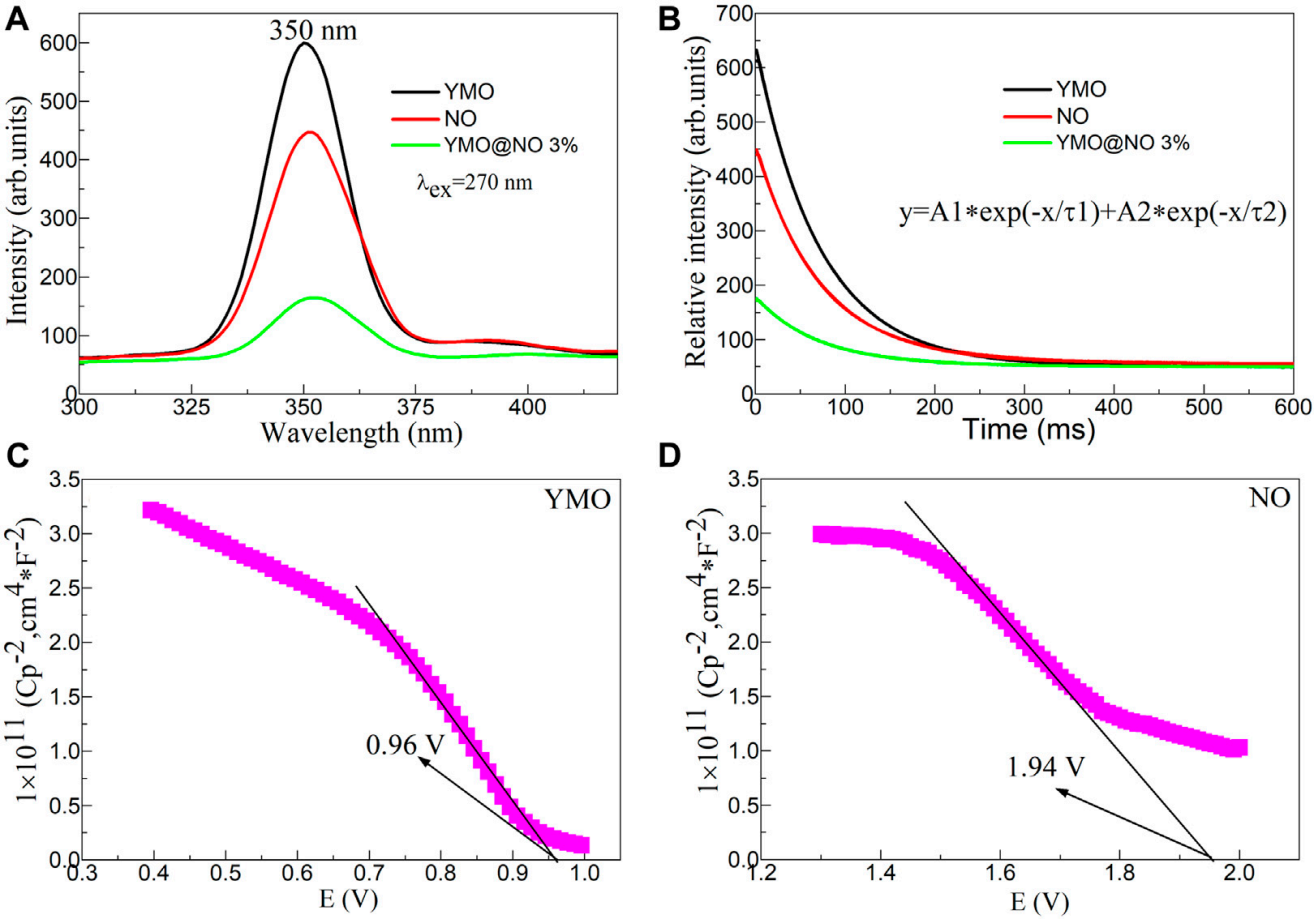
**(A)** Photoluminescence (PL) spectra excited at 270 nm, and **(B)** time-resolved PL (TRPL) spectra of the YMO, NO, and YMO@NO 3% photocatalysts. The Mott- Schottky (M–S) plots of **(C)** YMO and **(D)** NO.

By studying the photoluminescence properties, the transfer and separation efficiency of electron hole pairs of photocatalysts can be explored, which provides experimental support for further exploration of the photocatalytic mechanism of photocatalysts (He et al., 2022). Figure 9A shows the photoluminescence (PL) spectra excited at 270 nm of the YMO, NO, and YMO@NO 3% photocatalysts. An obvious emission peak at 350 nm can be observed. This emission peak is consistent with the results reported in the literature (Ahmad et al., 2015). The peak at 350 nm can be ascribed to the interatomic transition of Mn^3+^ (Miura et al., 2021; Munisha et al., 2023b). Notably, similar emission peaks were observed in NO. The emission peak at 350 nm for the NO can be assigned to a near band-edge emission (Anandan and Rajendran, 2011). When YMO and NO are coupled, YMO@NO 3% exhibits weak photoluminescence properties. The results show that YMO@NO 3% photocatalyst has high charge transfer and separation efficiency.

To further confirm this conclusion, Figure 9B shows the time-resolved PL (TRPL) spectra of the YMO, NO, and YMO@NO 3% photocatalysts. The initial luminous intensity corresponds to the maximum intensity value of the emission spectrum. TRPL can be simulated by double exponential model (Eq. (2)).

$$y=A_{1}*e^{\frac{-t}{\tau_{1}}}+A_{2}*e^{\frac{-t}{\tau_{2}}}$$ (2)

Where, y is the intensity, (A_1_, A_2_) is the initial intensity, t is the time, and (τ_1_, τ_2_) is the lifetime. The double exponential model is used to simulate TRPL, which is in good agreement with the experimental curve. Table 1 shows the fitted fluorescence lifetimes of the YMO, NO, and YMO@NO 3% photocatalysts. The YMO@NO 3% photocatalyst exhibits poor properties in terms of initial emission intensity and fluorescence lifetime, indicating that it has high charge transfer and separation efficiency. This conclusion is consistent with the characterization of PL properties.

**TABLE 1:** The fitted fluorescence lifetimes of the YMO, NO, and YMO@NO 3% photocatalysts.

Sample	A1	τ1 (ns)	A2	τ2 (ns)
YMO	588.02	71.80	5.93	802.21
NO	394.42	72.03	12.92	1199.57
YMO@NO 3%	124.95	70.88	5.98	986.64

Simultaneously, Mott- Schottky (M-S) curves of YMO and NO can be obtained through an electrochemical workstation (He et al., 2023a). The M-S plots of YMO and NO as shown in Figures 9C, D. The negative slope indicates that YMO and NO are p-type semiconductors. This result is consistent with the results reported in the literature (Ren et al., 2013; Ma et al., 2022). Based on Figures 9C, D, the estimated V_FB_ values were 0.96, and 1.94 V vs. SCE for the YMO and NO, respectively. The valence band potential for the p-type semiconductors at normal hydrogen electrode (NHE) can be expressed by the following Formula (3):

$$V\left(\text{NHE}\right)=V_{\text{FB}}+0.059\text{pH}+0.242$$ (3)

Where, pH = 8. The valence band potentials of YMO and NO are 1.674 and 2.654 V, respectively. These results will be helpful to further study of the mechanism of YMO@NO 3% photocatalyst.

### 3.10 Photocatalytic mechanism

It can be seen from the results of photocatalysis experiments that the YMO@NO photocatalyst exhibits high photocatalytic activity for the degradation of oil and gas field wastewater under simulated solar irradiation. Formulas 4, 5 are used to calculate the conduction band potential (*E*_CB_) and valence band potential (*E*_VB_) of YMO and NO in accordance with the energy band theory (He et al., 2023b; He et al., 2023c; He et al., 2024).

$$E_{\text{CB}}=X\;-\;E^{e}\;-\;0.5E_{g}$$ (4)

$$E_{\text{VB}}=E_{\text{CB}}+E_{g}$$ (5)

Where, *E* ^e^ = 4.5 eV is the energy of free electrons on the hydrogen scale. According to the literature (Thota and Kumar, 2007; Deng et al., 2009; Liu Y. et al., 2021) and the calculation in this experiment, the E.g., values of YMO and NO are 1.30 and 2.74 eV, respectively. Eqs 6, 7 were used to estimate the X values of the YMO and NO as 5.52 and 5.76 V, respectively.

$$X\left(NiO\right)=\sqrt[{2}]{X\left(Ni\right)X\left(O\right)}$$ (6)

$$X\left(YMnO_{3}\right)=\sqrt[{5}]{X\left(Y\right)X\left(Mn\right)X{\left(O\right)}^{3}}$$ (7)

**FIGURE 10:**
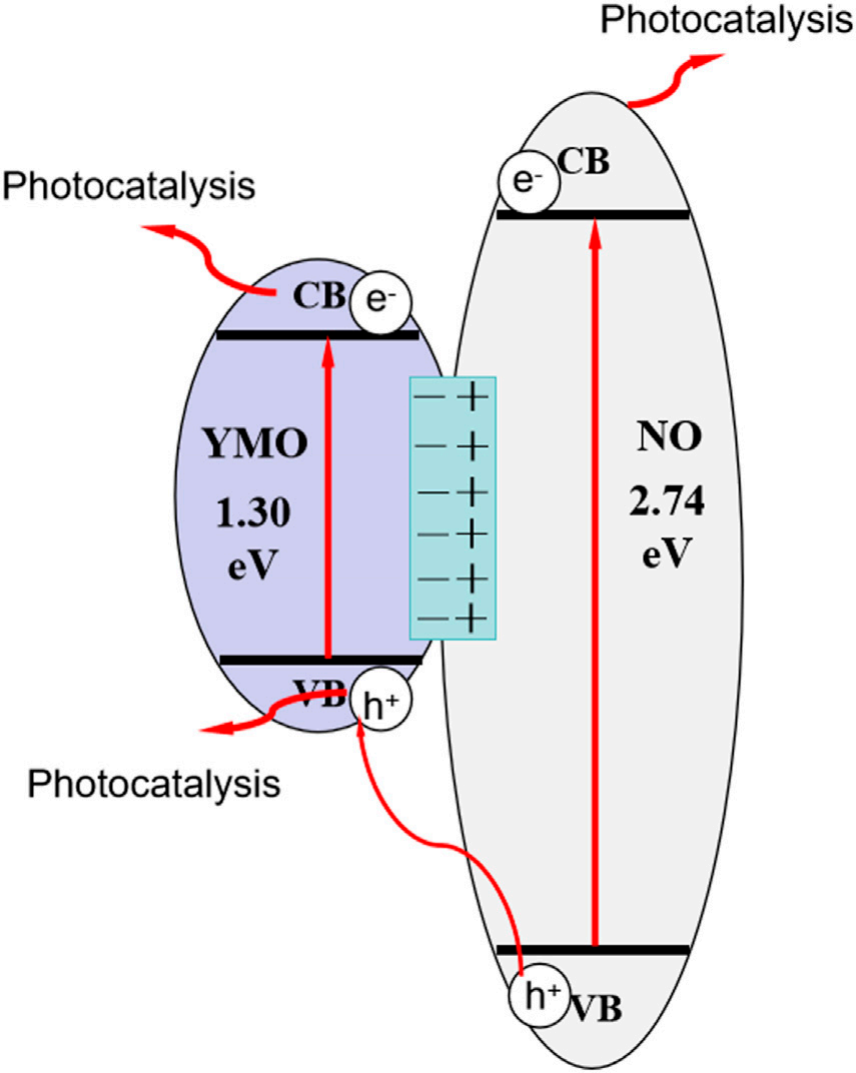
Photocatalytic mechanism of YMO@NO.

The values are 4.40 eV for X (Ni), 3.19 eV for X Y), 3.75 eV for X (Mn) and 7.54 eV for X O). YMO and NO have an *E*_CB_ of 0.37 and −0.11 V in their respective oxides. Both YMO and NO have an *E*_VB_ between 1.67 and 2.63 V. The result is similar to that obtained by M-S calculation. According to the above calculation, the energy level diagrams of YMO and NO can be drawn as depicted in Figure 10. A type I band arrangement is formed between YMO and NO. Generally, the type I band arrangement of heterojunction promotes the recombination rate of charge carrier, which is unfavorable to the photocatalytic degradation of pollutants. However, the YMO@NO photocatalyst synthesized in this study showed high photocatalytic activity. According to the literature (Yang, 2021), the high photocatalytic activity of the type I band arranged heterojunction is mainly due to the formation of an energy barrier at the interface of the two semiconductors, which will contribute to the effective separation of electrons and holes, thereby increasing the photocatalytic activity of the system. In this experiment, the energy barrier is successfully formed at the interface of YMO and NO. When the simulated sunlight shines on the surface of YMO@NO photocatalyst, the electrons of YMO and NO valence bands can be excited to transition to their respective conduction bands because the simulated sunlight contains ultraviolet light. This process can be described as following formulas (8), (9):

$$\text{YMO}+h\nu \;\to \text{ YMO }\left(e_{\text{CB}}^{-}+h_{\text{VB}}^{+}\right)$$ (8)

$$\text{NO}+h\nu \;\to \text{ NO }\left(e_{\text{CB}}^{-}+h_{\text{VB}}^{+}\right)$$ (9)

The reaction solution can produce superoxide radical (•O_2_^−^) by reacting the conduction band electron of NO with O_2_, which is evidenced by the fact that it has an redox potential is 0.13 V for O_2_/•O^2−^. The electrons of YMO conduction band are prevented from jumping to the conduction band of NO due to the action of the energy barrier. Hydroxyl radical (•OH) can be generated by YMO’s free conduction-band electron reacting with O_2_/H_2_O_2_ (0.695 V). The above process can be described as following formulas (10)–(14):

$$\text{NO}:e_{\text{CB}}^{-}+O_{2}\to •O^{2-}$$ (10)

$$\text{NO}:•O^{2-}+2H^{+}+e_{\text{CB}}^{-}\to H_{2}O_{2}$$ (11)

$$\text{YMO}:2e_{\text{CB}}^{-}+O_{2}+2H^{+}\;\to \;H_{2}O_{2}$$ (12)

$$\text{YMO}:e_{\text{CB}}^{-}+H_{2}O_{2}\;\to \;•\text{OH}+{\text{OH}}^{-}$$ (13)

$$\text{YMO}:•O^{2-}+H_{2}O_{2}\to •\text{OH}+{\text{OH}}^{-}+O_{2}$$ (14)

The hydroxyl radical and superoxide radical produced will react with pollutants to form non-toxic and harmless small molecular organic matter (Eq. (15)).

$$•\text{OH}+•O_{2}^{-}+\text{Pollutant }\to \text{ Degradation product}$$ (15)

The valence band potential of NO is more positive than that of YMO, which makes the NO hole jump to the valence band of YMO. However, the valence band hole of YMO cannot react with H_2_O to produce •OH radicals due to the redox potentials of H_2_O/•OH and OH^−^/•OH are +2.72 and +1.89 V *versus* NHE, respectively. This is also the main reason why hydroxyl radicals have been observed in the capture experiment to play a weak role in the entire photocatalytic process. It is worth noting that some conduction band electrons and valence band holes will directly react with pollutants to produce non-toxic and harmless products (Eqs (16), (17)).

$$e_{\text{CB}}^{-}+\text{Pollutant }\to \text{ Degradation product}$$ (16)

$$h_{\text{VB}}^{+}+\text{Pollutant }\to \text{ degradation products}$$ (17)

## 4 Conclusion

The YMO@NO photocatalysts with high photocatalytic activity for the degradation of oil and gas field wastewater under simulated solar irradiation were synthesized by one-step hydrothermal method with the urea as surfactant. XRD, FTIR and XPS confirm that the YMO@NO photocatalyst is free of any oxide impurities except YMO and NO. The YMO@NO photocatalysts consists of large irregular cuboids and fine spherical particles. The YMO@NO photocatalysts demonstrate high optical absorption coefficient and photocatalytic activity. When the catalyst content is 1.5 g/L, the mass percentage of NO is 3% and the irradiation time is 60 min, the degradation percentage of YMO@NO photocatalyst is 98%. Stability experiment, capture experiment and photocatalytic mechanism analysis show that the YMO@NO photocatalyst can be recycled, and electrons, holes, hydroxyl radicals and superoxide radicals are the main active species for the degrading of oil and gas field wastewater.

## Data Availability

The raw data supporting the conclusion of this article will be made available by the authors, without undue reservation.
